# 2,4-Dichloro-6-((1*R*)-1-{[(*R*)-(2-chloro­phen­yl)(cyclo­pent­yl)meth­yl]amino}eth­yl)phenol

**DOI:** 10.1107/S1600536809041403

**Published:** 2009-10-17

**Authors:** Guang-You Zhang, Di-Juan Chen, Shu-Hong Wang, Ting Yang, Jian-Guo Chang

**Affiliations:** a School of Chemistry and Chemical Engineering, University of Jinan, Shandong 250022, People’s Republic of China; b Jincheng Pharmaceutical Co Ltd, Shandong Provience, Shandong 255100, People’s Republic of China; c Department of Materials Science and Chemical Engineering, Taishan University, Shandong 271021, People’s Republic of China

## Abstract

In the title compound, C_20_H_22_Cl_3_NO, the five-membered ring adopts an envelope conformation, and the two benzene rings are oriented at a dihedral angle of 40.44 (9)°. Intra­molecular O—H⋯N and N—H⋯Cl hydrogen bonding is present. In the crystal, the mol­ecules are linked *via* weak inter­molecular C—H⋯O hydrogen bonds.

## Related literature

For amino­phenols, see: Li *et al.* (2004 ▶); Puigjaner *et al.* (1999 ▶); Cimarelli *et al.* (2002 ▶); Joshi & Malhotra (2003 ▶); Zhang *et al.* (2003 ▶); Watts *et al.* (2005 ▶). For the synthesis, see: Yang *et al.* (2005 ▶).
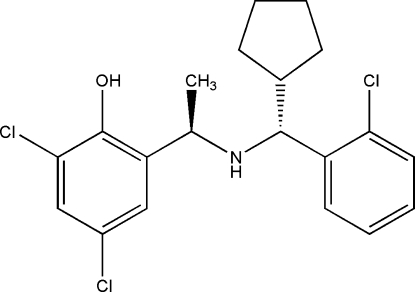


## Experimental

### 

#### Crystal data

C_20_H_22_Cl_3_NO
*M* *_r_* = 398.74
Orthorhombic, 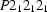

*a* = 8.4132 (7) Å
*b* = 13.6767 (10) Å
*c* = 17.0018 (14) Å
*V* = 1956.3 (3) Å^3^
*Z* = 4
Mo *K*α radiation
μ = 0.48 mm^−1^
*T* = 298 K
0.21 × 0.16 × 0.12 mm

#### Data collection

Bruker SMART CCD area-detector diffractometer
Absorption correction: multi-scan (*SADABS*; Sheldrick, 1996 ▶) *T*_min_ = 0.907, *T*_max_ = 0.945
10361 measured reflections
3453 independent reflections
3005 reflections with *I* > 2σ(*I*)
*R*_int_ = 0.022

#### Refinement

*R*[*F* ^2^ > 2σ(*F* ^2^)] = 0.036
*wR*(*F* ^2^) = 0.096
*S* = 1.04
3453 reflections
227 parameters
H-atom parameters constrained
Δρ_max_ = 0.20 e Å^−3^
Δρ_min_ = −0.18 e Å^−3^
Absolute structure: Flack (1983 ▶), 1464 Friedel pairs
Flack parameter: 0.00 (7)

### 

Data collection: *SMART* (Bruker, 1999 ▶); cell refinement: *SAINT* (Bruker, 1999 ▶); data reduction: *SAINT*; program(s) used to solve structure: *SHELXTL* (Sheldrick, 2008 ▶); program(s) used to refine structure: *SHELXTL*; molecular graphics: *SHELXTL*; software used to prepare material for publication: *SHELXTL*.

## Supplementary Material

Crystal structure: contains datablocks I, global. DOI: 10.1107/S1600536809041403/xu2619sup1.cif

Structure factors: contains datablocks I. DOI: 10.1107/S1600536809041403/xu2619Isup2.hkl

Additional supplementary materials:  crystallographic information; 3D view; checkCIF report

## Figures and Tables

**Table 1:** Hydrogen-bond geometry (Å, °)

*D*—H⋯*A*	*D*—H	H⋯*A*	*D*⋯*A*	*D*—H⋯*A*
O1—H1*A*⋯N1	0.96	1.74	2.622 (3)	151
N1—H1*N*⋯Cl3	0.84	2.68	3.260 (2)	127
C13—H13⋯O1^i^	0.93	2.56	3.422 (3)	154

Symmetry code: (i) 
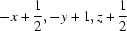
.

**Fig. 1.**
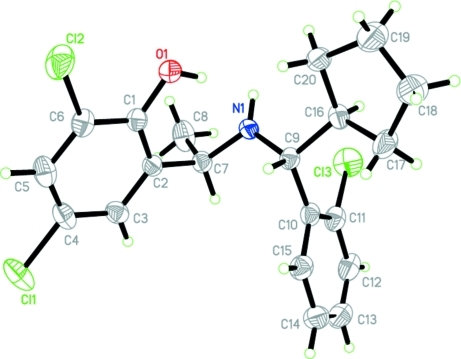
The structure of the title compound with 30% probability ellipsoids. H atoms are shown as spheres of arbitrary radii.

## supplementary crystallographic information

### Comment

The chiral aminophenols containing some O and N atoms are of great interests due to their widespread application in asymmetric synthesis such as chiral bases, auxiliaries and ligands (Li *et al.*, 2004; Puigjaner *et al.*, 1999; Cimarelli *et al.*, 2002). Recently, the synthesis of chiral aminophenols with a varity of functionalities has attracted increasing attention (Zhang *et al.*, 2003; Watts *et al.*, 2005). Herein, we present the molecular structure of the title aminophenol (I), which was initially prepared to test its catalytic activity. The aminophenol was prepared by conventional condensation of (*R*)-1-(2-chlorophenyl)-1-cyclopentylmethanamine with 1-(3,5-dichloro-2-hydroxyphenyl) ethanone in methanol.

The molecular structure of (I) is illustrated in Fig. 1. The title compoud has two chiral centers (C7/C9), which have configurations *R*, *R*, confirmed by the X-ray structural analysis. There are the intramolecular O—H···N and N—H···Cl hydrogen bonding which stablizes the conformation of the molecule (Table 1). In the crystal packing, the molecules are linked to each other *via* intermolecular C—H···O hydrogen bonds (Table 1).

### Experimental

The title compound was prepared according to the procedure of Yang *et al.* (2005). (*R*)-1-(2-chlorophenyl)-1-cyclopentylmethanamine (0.9 mmol) and 1-(3,5-dichloro-2-hydroxyphenyl)ethanone (0.9 mmol) were dissolved in methanol (10 ml) and reacted at room temperature for 48 h. After removal of the solvent, NaBH_4_ (4.5 mmol) was added to the solution in THF/ethanol (1:1 *v*/*v*, 20 ml) and stirred at 273 K until the solution became colourless. The solvent was then removed under reduced pressure. Water (10 ml) was added to the residue and 1 *M* HCl was added dropwise until hydrogen production ceased. The mixture was neutralized with aqueous solution of Na_2_CO_3_, then extracted with CHCl_3_, and the organic layer was dried over anhydrous sodium sulfate. The solvent was removed under reduced pressure. Further purification was carried out by thin-layer silica-gel chromatography (chloroform) to give a colorless solid (yield 82.7%). Single crystals of (I) were grown from the n-hexane solution.

### Refinement

Imino-H and hydroxy-H atoms were located in a difference Fourier map and refined as riding in as-found relative positions with *U*_iso_(H) = 1.2*U*_eq_(N,*O*). Other H atoms were placed in geometrically idealized positions and constrained to ride on their parent atoms with C—H = 0.93–0.98 Å, and refined in riding mode with *U*_iso_(H) = 1.5*U*_eq_(C) for mathyl H atoms and 1.2*U*_eq_(C) for the others.

### Crystal data

**Table:**

C_20_H_22_Cl_3_NO	*F*(000) = 832
*M**_r_* = 398.74	*D*_x_ = 1.354 Mg m^−^^3^
Orthorhombic, *P*2_1_2_1_2_1_	Mo *K*α radiation, λ = 0.71073 Å
Hall symbol: P 2ac 2ab	Cell parameters from 4018 reflections
*a* = 8.4132 (7) Å	θ = 2.4–23.8°
*b* = 13.6767 (10) Å	µ = 0.48 mm^−^^1^
*c* = 17.0018 (14) Å	*T* = 298 K
*V* = 1956.3 (3) Å^3^	Plate, colorless
*Z* = 4	0.21 × 0.16 × 0.12 mm

### Data collection

**Table:**

Bruker SMART CCD area-detector diffractometer	3453 independent reflections
Radiation source: fine-focus sealed tube	3005 reflections with *I* > 2σ(*I*)
graphite	*R*_int_ = 0.022
φ and ω scans	θ_max_ = 25.1°, θ_min_ = 1.9°
Absorption correction: multi-scan (*SADABS*; Sheldrick, 1996)	*h* = −9→10
*T*_min_ = 0.907, *T*_max_ = 0.945	*k* = −16→13
10361 measured reflections	*l* = −20→19

### Refinement

**Table:**

Refinement on *F*^2^	Secondary atom site location: difference Fourier map
Least-squares matrix: full	Hydrogen site location: inferred from neighbouring sites
*R*[*F*^2^ > 2σ(*F*^2^)] = 0.036	H-atom parameters constrained
*wR*(*F*^2^) = 0.096	*w* = 1/[σ^2^(*F*_o_^2^) + (0.0479*P*)^2^ + 0.4088*P*] where *P* = (*F*_o_^2^ + 2*F*_c_^2^)/3
*S* = 1.04	(Δ/σ)_max_ = 0.001
3453 reflections	Δρ_max_ = 0.20 e Å^−^^3^
227 parameters	Δρ_min_ = −0.18 e Å^−^^3^
0 restraints	Absolute structure: Flack (1983), 1464 Friedel pairs
Primary atom site location: structure-invariant direct methods	Flack parameter: 0.00 (7)

### Special details

**Table:**

Geometry. All e.s.d.'s (except the e.s.d. in the dihedral angle between two l.s. planes) are estimated using the full covariance matrix. The cell e.s.d.'s are taken into account individually in the estimation of e.s.d.'s in distances, angles and torsion angles; correlations between e.s.d.'s in cell parameters are only used when they are defined by crystal symmetry. An approximate (isotropic) treatment of cell e.s.d.'s is used for estimating e.s.d.'s involving l.s. planes.
Refinement. Refinement of *F*^2^ against ALL reflections. The weighted *R*-factor *wR* and goodness of fit *S* are based on *F*^2^, conventional *R*-factors *R* are based on *F*, with *F* set to zero for negative *F*^2^. The threshold expression of *F*^2^ > σ(*F*^2^) is used only for calculating *R*-factors(gt) *etc*. and is not relevant to the choice of reflections for refinement. *R*-factors based on *F*^2^ are statistically about twice as large as those based on *F*, and *R*- factors based on ALL data will be even larger.

### Fractional atomic coordinates and isotropic or equivalent isotropic displacement parameters (Å^2^)

**Table:**

	*x*	*y*	*z*	*U*_iso_*/*U*_eq_	
Cl1	0.74492 (12)	0.20053 (6)	0.65026 (5)	0.0892 (3)	
Cl2	0.77477 (11)	0.32755 (7)	0.35243 (5)	0.0818 (3)	
Cl3	0.17174 (12)	0.73428 (6)	0.60165 (5)	0.0812 (3)	
N1	0.4064 (2)	0.58218 (15)	0.51047 (11)	0.0438 (5)	
H1N	0.3999	0.6424	0.5201	0.053*	
O1	0.6034 (2)	0.49741 (14)	0.41237 (10)	0.0568 (5)	
H1A	0.5277	0.5427	0.4341	0.068*	
C1	0.6303 (3)	0.42963 (19)	0.46839 (14)	0.0444 (6)	
C2	0.5780 (3)	0.44142 (18)	0.54534 (14)	0.0421 (5)	
C3	0.6114 (3)	0.36909 (19)	0.60025 (16)	0.0498 (6)	
H3	0.5742	0.3755	0.6515	0.060*	
C4	0.6985 (3)	0.28820 (19)	0.57958 (18)	0.0568 (7)	
C5	0.7529 (4)	0.2752 (2)	0.50434 (17)	0.0603 (7)	
H5	0.8136	0.2208	0.4910	0.072*	
C6	0.7148 (3)	0.3454 (2)	0.44879 (15)	0.0529 (6)	
C7	0.5002 (3)	0.53592 (18)	0.57377 (14)	0.0464 (6)	
H7	0.4300	0.5211	0.6182	0.056*	
C8	0.6279 (4)	0.6086 (2)	0.6006 (2)	0.0712 (9)	
H8A	0.7007	0.6206	0.5582	0.107*	
H8B	0.6847	0.5818	0.6446	0.107*	
H8C	0.5784	0.6689	0.6159	0.107*	
C9	0.2437 (3)	0.54384 (17)	0.49936 (12)	0.0413 (5)	
H9	0.2565	0.4764	0.4809	0.050*	
C10	0.1458 (3)	0.53736 (19)	0.57434 (14)	0.0462 (6)	
C11	0.1105 (3)	0.6155 (2)	0.62414 (15)	0.0551 (7)	
C12	0.0257 (3)	0.6038 (3)	0.69294 (16)	0.0657 (9)	
H12	0.0036	0.6578	0.7243	0.079*	
C13	−0.0256 (4)	0.5139 (3)	0.71500 (17)	0.0722 (9)	
H13	−0.0807	0.5061	0.7620	0.087*	
C14	0.0042 (4)	0.4338 (3)	0.66735 (19)	0.0717 (9)	
H14	−0.0323	0.3722	0.6821	0.086*	
C15	0.0871 (3)	0.4449 (2)	0.59875 (16)	0.0555 (7)	
H15	0.1055	0.3905	0.5673	0.067*	
C16	0.1623 (3)	0.5990 (2)	0.43215 (14)	0.0480 (6)	
H16	0.1482	0.6675	0.4478	0.058*	
C17	−0.0009 (4)	0.5558 (3)	0.41129 (17)	0.0771 (10)	
H17A	−0.0032	0.4861	0.4214	0.092*	
H17B	−0.0843	0.5870	0.4417	0.092*	
C18	−0.0216 (4)	0.5764 (4)	0.32453 (19)	0.1024 (14)	
H18A	−0.0591	0.5182	0.2978	0.123*	
H18B	−0.0995	0.6279	0.3170	0.123*	
C19	0.1269 (4)	0.6057 (4)	0.29287 (18)	0.1004 (15)	
H19A	0.1208	0.6730	0.2752	0.121*	
H19B	0.1533	0.5650	0.2480	0.121*	
C20	0.2520 (3)	0.5960 (2)	0.35444 (14)	0.0607 (7)	
H20A	0.3275	0.6494	0.3511	0.073*	
H20B	0.3088	0.5346	0.3487	0.073*	

### Atomic displacement parameters (Å^2^)

**Table:**

	*U*^11^	*U*^22^	*U*^33^	*U*^12^	*U*^13^	*U*^23^
Cl1	0.1119 (7)	0.0569 (4)	0.0988 (6)	0.0057 (5)	−0.0350 (6)	0.0237 (4)
Cl2	0.0826 (6)	0.0970 (6)	0.0659 (5)	0.0243 (5)	0.0076 (4)	−0.0202 (4)
Cl3	0.0997 (6)	0.0587 (4)	0.0852 (5)	0.0023 (4)	0.0080 (5)	−0.0205 (4)
N1	0.0408 (10)	0.0424 (11)	0.0481 (11)	0.0033 (9)	−0.0060 (9)	0.0035 (9)
O1	0.0513 (10)	0.0707 (12)	0.0484 (10)	0.0118 (9)	0.0076 (8)	0.0129 (9)
C1	0.0368 (12)	0.0505 (14)	0.0458 (13)	0.0015 (11)	−0.0041 (10)	0.0016 (11)
C2	0.0354 (12)	0.0429 (13)	0.0480 (14)	−0.0021 (11)	−0.0048 (10)	0.0026 (11)
C3	0.0511 (14)	0.0501 (14)	0.0483 (14)	−0.0045 (12)	−0.0077 (12)	0.0047 (11)
C4	0.0573 (16)	0.0423 (15)	0.0709 (19)	0.0000 (12)	−0.0216 (14)	0.0066 (13)
C5	0.0590 (16)	0.0455 (15)	0.077 (2)	0.0077 (14)	−0.0160 (14)	−0.0090 (13)
C6	0.0455 (14)	0.0606 (16)	0.0527 (15)	0.0038 (13)	−0.0047 (12)	−0.0112 (12)
C7	0.0444 (12)	0.0514 (15)	0.0433 (13)	0.0056 (12)	−0.0034 (11)	0.0009 (11)
C8	0.0663 (19)	0.0613 (18)	0.086 (2)	0.0053 (15)	−0.0273 (17)	−0.0162 (16)
C9	0.0398 (12)	0.0444 (12)	0.0397 (12)	0.0040 (11)	0.0000 (10)	−0.0036 (9)
C10	0.0376 (12)	0.0568 (15)	0.0442 (13)	0.0039 (11)	−0.0060 (10)	0.0000 (11)
C11	0.0459 (14)	0.0722 (18)	0.0473 (14)	0.0037 (13)	−0.0065 (12)	−0.0100 (13)
C12	0.0479 (16)	0.107 (3)	0.0423 (15)	0.0089 (17)	−0.0037 (13)	−0.0125 (16)
C13	0.0513 (17)	0.121 (3)	0.0446 (16)	0.0014 (19)	−0.0023 (13)	0.0113 (19)
C14	0.0548 (16)	0.091 (2)	0.069 (2)	−0.0134 (18)	−0.0066 (16)	0.0246 (18)
C15	0.0467 (14)	0.0685 (18)	0.0512 (15)	−0.0014 (13)	−0.0035 (12)	−0.0006 (13)
C16	0.0487 (14)	0.0514 (15)	0.0438 (13)	0.0091 (12)	−0.0049 (11)	−0.0032 (11)
C17	0.0420 (14)	0.137 (3)	0.0519 (17)	0.0015 (19)	−0.0033 (13)	0.0090 (18)
C18	0.062 (2)	0.189 (4)	0.0560 (19)	−0.016 (3)	−0.0167 (16)	0.025 (2)
C19	0.064 (2)	0.192 (5)	0.0453 (17)	0.001 (3)	−0.0046 (15)	0.014 (2)
C20	0.0469 (15)	0.088 (2)	0.0476 (14)	−0.0023 (14)	−0.0027 (13)	0.0102 (14)

### Geometric parameters (Å, °)

**Table:**

Cl1—C4	1.742 (3)	C10—C11	1.395 (4)
Cl2—C6	1.731 (3)	C10—C15	1.419 (4)
Cl3—C11	1.747 (3)	C11—C12	1.379 (4)
N1—C7	1.477 (3)	C12—C13	1.356 (5)
N1—C9	1.478 (3)	C12—H12	0.9300
N1—H1N	0.8410	C13—C14	1.386 (5)
O1—C1	1.348 (3)	C13—H13	0.9300
O1—H1A	0.9621	C14—C15	1.368 (4)
C1—C2	1.390 (4)	C14—H14	0.9300
C1—C6	1.395 (4)	C15—H15	0.9300
C2—C3	1.389 (3)	C16—C20	1.522 (3)
C2—C7	1.527 (3)	C16—C17	1.536 (4)
C3—C4	1.373 (4)	C16—H16	0.9800
C3—H3	0.9300	C17—C18	1.512 (4)
C4—C5	1.370 (4)	C17—H17A	0.9700
C5—C6	1.384 (4)	C17—H17B	0.9700
C5—H5	0.9300	C18—C19	1.417 (5)
C7—C8	1.533 (4)	C18—H18A	0.9700
C7—H7	0.9800	C18—H18B	0.9700
C8—H8A	0.9600	C19—C20	1.491 (4)
C8—H8B	0.9600	C19—H19A	0.9700
C8—H8C	0.9600	C19—H19B	0.9700
C9—C10	1.521 (3)	C20—H20A	0.9700
C9—C16	1.531 (3)	C20—H20B	0.9700
C9—H9	0.9800		
			
C7—N1—C9	115.86 (19)	C12—C11—Cl3	116.5 (2)
C7—N1—H1N	108.2	C10—C11—Cl3	121.1 (2)
C9—N1—H1N	108.2	C13—C12—C11	120.2 (3)
C1—O1—H1A	106.4	C13—C12—H12	119.9
O1—C1—C2	122.1 (2)	C11—C12—H12	119.9
O1—C1—C6	119.0 (2)	C12—C13—C14	119.9 (3)
C2—C1—C6	118.8 (2)	C12—C13—H13	120.1
C3—C2—C1	119.1 (2)	C14—C13—H13	120.1
C3—C2—C7	118.5 (2)	C15—C14—C13	120.1 (3)
C1—C2—C7	122.1 (2)	C15—C14—H14	119.9
C4—C3—C2	120.6 (3)	C13—C14—H14	119.9
C4—C3—H3	119.7	C14—C15—C10	121.8 (3)
C2—C3—H3	119.7	C14—C15—H15	119.1
C5—C4—C3	121.5 (2)	C10—C15—H15	119.1
C5—C4—Cl1	118.7 (2)	C20—C16—C9	114.3 (2)
C3—C4—Cl1	119.8 (2)	C20—C16—C17	103.4 (2)
C4—C5—C6	118.0 (2)	C9—C16—C17	112.5 (2)
C4—C5—H5	121.0	C20—C16—H16	108.8
C6—C5—H5	121.0	C9—C16—H16	108.8
C5—C6—C1	121.9 (2)	C17—C16—H16	108.8
C5—C6—Cl2	118.7 (2)	C18—C17—C16	104.8 (3)
C1—C6—Cl2	119.4 (2)	C18—C17—H17A	110.8
N1—C7—C2	111.16 (19)	C16—C17—H17A	110.8
N1—C7—C8	108.3 (2)	C18—C17—H17B	110.8
C2—C7—C8	110.0 (2)	C16—C17—H17B	110.8
N1—C7—H7	109.1	H17A—C17—H17B	108.9
C2—C7—H7	109.1	C19—C18—C17	108.8 (3)
C8—C7—H7	109.1	C19—C18—H18A	109.9
C7—C8—H8A	109.5	C17—C18—H18A	109.9
C7—C8—H8B	109.5	C19—C18—H18B	109.9
H8A—C8—H8B	109.5	C17—C18—H18B	109.9
C7—C8—H8C	109.5	H18A—C18—H18B	108.3
H8A—C8—H8C	109.5	C18—C19—C20	109.3 (3)
H8B—C8—H8C	109.5	C18—C19—H19A	109.8
N1—C9—C10	114.55 (18)	C20—C19—H19A	109.8
N1—C9—C16	109.57 (19)	C18—C19—H19B	109.8
C10—C9—C16	114.31 (19)	C20—C19—H19B	109.8
N1—C9—H9	105.9	H19A—C19—H19B	108.3
C10—C9—H9	105.9	C19—C20—C16	104.9 (2)
C16—C9—H9	105.9	C19—C20—H20A	110.8
C11—C10—C15	115.5 (2)	C16—C20—H20A	110.8
C11—C10—C9	125.4 (2)	C19—C20—H20B	110.8
C15—C10—C9	119.1 (2)	C16—C20—H20B	110.8
C12—C11—C10	122.4 (3)	H20A—C20—H20B	108.8
			
O1—C1—C2—C3	−179.4 (2)	C16—C9—C10—C11	−69.6 (3)
C6—C1—C2—C3	0.0 (4)	N1—C9—C10—C15	−120.1 (2)
O1—C1—C2—C7	−5.9 (4)	C16—C9—C10—C15	112.3 (3)
C6—C1—C2—C7	173.5 (2)	C15—C10—C11—C12	0.6 (4)
C1—C2—C3—C4	1.8 (4)	C9—C10—C11—C12	−177.5 (2)
C7—C2—C3—C4	−171.9 (2)	C15—C10—C11—Cl3	−179.05 (19)
C2—C3—C4—C5	−1.2 (4)	C9—C10—C11—Cl3	2.8 (3)
C2—C3—C4—Cl1	177.7 (2)	C10—C11—C12—C13	0.7 (4)
C3—C4—C5—C6	−1.2 (4)	Cl3—C11—C12—C13	−179.6 (2)
Cl1—C4—C5—C6	179.9 (2)	C11—C12—C13—C14	−1.5 (4)
C4—C5—C6—C1	3.1 (4)	C12—C13—C14—C15	0.9 (4)
C4—C5—C6—Cl2	−177.1 (2)	C13—C14—C15—C10	0.4 (4)
O1—C1—C6—C5	176.9 (2)	C11—C10—C15—C14	−1.2 (4)
C2—C1—C6—C5	−2.4 (4)	C9—C10—C15—C14	177.1 (2)
O1—C1—C6—Cl2	−2.9 (3)	N1—C9—C16—C20	56.3 (3)
C2—C1—C6—Cl2	177.76 (19)	C10—C9—C16—C20	−173.6 (2)
C9—N1—C7—C2	83.1 (2)	N1—C9—C16—C17	173.9 (2)
C9—N1—C7—C8	−155.9 (2)	C10—C9—C16—C17	−56.0 (3)
C3—C2—C7—N1	−153.0 (2)	C20—C16—C17—C18	−26.1 (4)
C1—C2—C7—N1	33.4 (3)	C9—C16—C17—C18	−150.0 (3)
C3—C2—C7—C8	87.0 (3)	C16—C17—C18—C19	13.3 (5)
C1—C2—C7—C8	−86.5 (3)	C17—C18—C19—C20	5.6 (6)
C7—N1—C9—C10	49.9 (3)	C18—C19—C20—C16	−22.4 (5)
C7—N1—C9—C16	179.88 (18)	C9—C16—C20—C19	152.1 (3)
N1—C9—C10—C11	58.0 (3)	C17—C16—C20—C19	29.4 (4)

### Hydrogen-bond geometry (Å, °)

**Table:**

*D*—H···*A*	*D*—H	H···*A*	*D*···*A*	*D*—H···*A*
O1—H1A···N1	0.96	1.74	2.622 (3)	151
N1—H1N···Cl3	0.84	2.68	3.260 (2)	127
C13—H13···O1^i^	0.93	2.56	3.422 (3)	154

Symmetry codes: (i) −*x*+1/2, −*y*+1, *z*+1/2.
